# A comparative ultrastructural and molecular biological study on *Chlamydia psittaci *infection in alpha-1 antitrypsin deficiency and non-alpha-1 antitrypsin deficiency emphysema versus lung tissue of patients with hamartochondroma

**DOI:** 10.1186/1471-2334-4-38

**Published:** 2004-09-21

**Authors:** Dirk Theegarten, Olaf Anhenn, Helmut Hotzel, Mathias Wagner, Alessandro Marra, Georgios Stamatis, Grigori Mogilevski, Konrad Sachse

**Affiliations:** 1 Institute of Pathology, Ruhr University Bochum, BG-Kliniken Bergmannsheil, Bürkle-de-la-Camp-Platz 1, D-44789 Bochum, Germany; 2 Institute of Bacterial Infections and Zoonoses, Friedrich-Loeffler-Institute (FLI), Federal Research Institute for Animal Health, Naumburger Strasse 96 a, D-07743 Jena, Germany; 3 Department of Thoracic Surgery, Ruhrlandklinik, Tüschener Weg 40, D-45239 Essen, Germany

## Abstract

**Background:**

*Chlamydiales* are familiar causes of acute and chronic infections in humans and animals. Human pulmonary emphysema is a component of chronic obstructive pulmonary disease (COPD) and a condition in which chronic inflammation manifested as bronchiolitis and intra-alveolar accumulation of macrophages is common. It is generally presumed to be of infectious origin. Previous investigations based on serology and immunohistochemistry indicated *Chlamydophila pneumoniae* infection in cases of COPD. Furthermore, immunofluorescence with genus-specific antibodies and electron microscopy suggested involvement of chlamydial infection in most cases of pulmonary emphysema, but these findings could not be verified by PCR. Therefore, we examined the possibility of other chlamydial species being present in these patients.

**Methods:**

Tissue samples from patients having undergone lung volume reduction surgery for advanced alpha-1 antitrypsin deficiency (AATD, n = 6) or non-alpha-1 antitrypsin deficiency emphysema (n = 34) or wedge resection for hamartochondroma (n = 14) were examined by transmission electron microscopy and PCR.

**Results:**

In all cases of AATD and 79.4% of non-AATD, persistent chlamydial infection was detected by ultrastructural examination. Intra-alveolar accumulation of macrophages and acute as well as chronic bronchiolitis were seen in all positive cases. The presence of *Chlamydia psittaci *was demonstrated by PCR in lung tissue of 66.7% AATD vs. 29.0% non-AATD emphysema patients. Partial DNA sequencing of four positive samples confirmed the identity of the agent as *Chlamydophila psittaci*. In contrast, *Chlamydophila pneumoniae *was detected only in one AATD patient. Lung tissue of the control group of non-smokers with hamartochondroma was completely negative for chlamydial bodies by TEM or chlamydial DNA by PCR.

**Conclusions:**

These data indicate a role of *Chlamydophila psittaci *in pulmonary emphysema by linking this chronic inflammatory process to a chronic infectious condition. This raises interesting questions on pathogenesis and source of infection.

## Background

Several species of the family *Chlamydiaceae *are well-known etiological agents of acute and chronic infections in humans and animals [1,2]. The first description of chlamydial respiratory disease in humans referred to psittacosis, also known as ornithosis, and dates back to 1879 [3]. *Chlamydia (C.) psittaci*, the agent responsible for this disease, has had several different names and, according to a recent proposal, should now be called *Chlamydophila (Cp.) psittaci *[4]. A century later, in 1986, Grayston et al. discovered another chlamydial respiratory agent, strain TWAR, which was later assigned to the species *C. pneumoniae *[5,6] currently reclassified as *Chlamydophila pneumoniae *[4]. Meanwhile, a variety of respiratory conditions in humans has been shown to be associated with this agent. Evidence of *Cp. pneumoniae *infection based on serology was reported in severe cases of chronic obstructive pulmonary disease (COPD), in which emphysema is dominant [7,8], as well as in exacerbations of COPD [9] and in persistent infections of the respiratory tract [10,11]. The detection rate of *Cp. pneumoniae *by immunohistochemical staining was elevated in lung tissue from subjects with COPD, but controls were not completely negative [12]. Initially *Cp. pneumoniae *was thought to be virulent for humans only, but recent descriptions of isolates from horse, koala, frog and reptiles suggest a wider host spectrum and even the possibility of zoonotic transmission [13-15].

Our previous investigations by means of immunofluorescence using a genus-specific antiserum against chlamydial LPS and scanning as well as transmission electron microscopy showed infection of the alveolar parenchyma and the bronchioles by *Chlamydia spp. *in patients having undergone lung volume reduction surgery for advanced pulmonary emphysema [16,17]. Accumulation of alveolar macrophages as well as different forms of bronchiolitis and focal pneumonia accompanying emphysematic changes were found regularly [18]. In preliminary examinations using an established nested PCR with DNA hybridization [19], DNA specific of *Cp. pneumoniae *was detected in two out of ten cases [20]. But this detection rate was far lower than that in electron microscopy or immunofluorescence using genus-specific antibodies, which showed *Chlamydia spp. *in over 80% [17]. Because of this fact PCR was extended to other *Chlamydiaceae*. Here we report the results of a more detailed study involving a larger number of cases and samples including controls.

## Methods

### Samples

Lung tissue of adequate quality from patients with advanced emphysema undergoing lung volume reduction surgery was used for the present study [18,21]. Samples examined by transmission electron microscopy (TEM) included five specimens from alpha-1 antitrypsin deficiency (AATD) and 34 from non-AATD patients. PCR examinations were conducted on six AATD and 31 non-AATD specimens. History showed a status of cigarette smoking with over ten packyears in most of these patients (91.7%). There were two non-smokers among nine patients with AATD. Furthermore patients with hamartochondroma undergoing wedge resection were reviewed for clinical data (A.M.) and histology. Normal lung tissue of 14 non-smokers taken with resection of hamartochondroma was selected as a control group. Statistical analysis was done using SPSS, version 11.5 (SPSS Inc., Chicago, USA) on a PC running Windows XP Professional (Microsoft, Redmond, USA) as operating system. A test value below 0.05 was considered to be statistically significant.

### Light Microscopy and Transmission Electron Microscopy (TEM)

Formalin-fixed lung tissue was embedded in paraffin wax (Tissuewax™; Medite GmbH, Burgdorf, Germany), slides of 3–7 μm thickness were cut using a rotatory microtome (Microm GmbH, Walldorf, Germany) and stained by hematoxylin and eosin. For TEM, tissue was fixed in 2.5% buffered glutaraldehyde or 3.5% formaldehyde and embedded in epon after postfixation with osmium tetroxide and block contrastation with uranyl acetate. In the cases of the hamartochrondroma control group, cores with diameters of 0.60 cm and 0.24 cm were obtained from paraffin blocks using a prototypical self-made manual tissue puncher (a device for tissue microarray construction developed by M.W.). These cores were used to select an area well defined by light microscopy for PCR (0.60 cm cores) and TEM (0.24 cm cores). For TEM, tissue was dewaxed with xylene and processed as described above. Semithin sections (prepared on a Reichert Om U3 ultramicrotome; Reichert, Vienna, Austria) were stained with basic fuchsin and methylene blue to define blocks of adequate quality. Ultrathin sections from two to five blocks were stained with lead citrate and examined using a Zeiss EM 900 transmission electron microscope (Zeiss, Oberkochen, Germany).

### Polymerase Chain Reaction (PCR)

Tissue from two different resources was used. Firstly, frozen tissue was collected immediately after resection and stored at -80°C (n = 31). Secondly, paraffin-embedded tissue (PET) containing histologically discernible inflammation sites was used, and sections or 0.60 cm cores were dewaxed using xylene (n = 12 and 14 controls). In five cases, material was available as both frozen and PET. DNA was isolated from lung tissue using the High Pure PCR Template Preparation Kit (Roche Diagnostics, Mannheim, Germany) according to the instructions of the manufacturer. Five μl of the DNA extract were used as template in PCR.

Samples were tested for*C. psittaci *and *Cp. pneumoniae *by a modified version of the nested PCR procedure described by Kaltenböck et al. [22], which targets the *omp*A gene. The first step was genus-specific amplification using primers 191CHOMP (5'-GCI YTI TGG GAR TGY GGI TGY GCI AC-3') and CHOMP371 (5'-TTA GAA ICK GAA TTG IGC RTT IAY GTG IGC IGC-3'). For the second amplification, we used 1 μl of the genus-specific product and primer combination 218PSITT (5'-GTA ATT TCI AGC CCA GCA CAA TTY GTG-3') / CHOMP336s (5'-CCR CAA GMT TTT CTR GAY TTC AWY TTG TTR AT-3') for *C. psittaci*, or 201CHOMP (5'-GGI GCW GMI TTC CAA TAY GCI CAR TC-3') / PNEUM268 (5'-GTA CTC CAA TGT ATG GCA CTA AAG A-3'), for *Cp. pneumoniae*, respectively. The sizes of specific amplicons are: 576–597 bp (genus-specific product), and 389–404 bp for *C. psittaci *or 244 bp for *Cp. pneumoniae *after nested PCR. A detailed protocol of the procedure is contained in [23].

### DNA sequencing

In order to discriminate the different members of the *C. psittaci*-group [4], five μl of the final DNA extract served as template for PCR amplification of the 16S rRNA signature region. Amplicon bands were cut out of agarose gels, extracted using the QIAquick Gel Extraction Kit (QIAGEN, Hilden, Germany), and subjected to cycle sequencing using the BigDye™ Terminator Cycle Sequencing Ready Reaction Kit (Applied Biosystems, Darmstadt, Germany). The oligonucleotides 16SIGF (5'-CCG CGT GGA TGA GGC AT-3') and 16SIGR (5'-TCA GTC CCA GTG TTG GC-3') were used as amplification and sequencing primers [4]. Nucleotide sequences were determined on an ABI Prism 310 Genetic Analyzer (Applied Biosystems).

## Results

### Light microscopy

**Figure 1:**
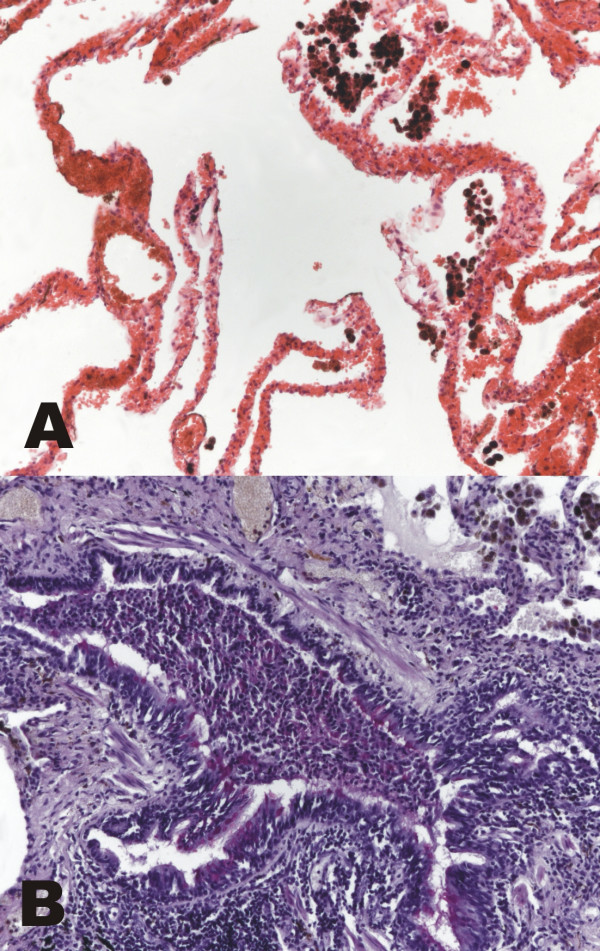
**Histology of alpha-1 antitrypsin deficiency emphysema. **In alpha-1 antitrypsin deficiency, advanced panacinar destruction of the lung parenchyma and accumulation of macrophages (A, hematoxylin eosin, original magnification ×40), as well as severe acute and chronic bronchiolitis are seen (B, periodic acid Schiff's reaction, original magnification ×100).

**Figure 2:**
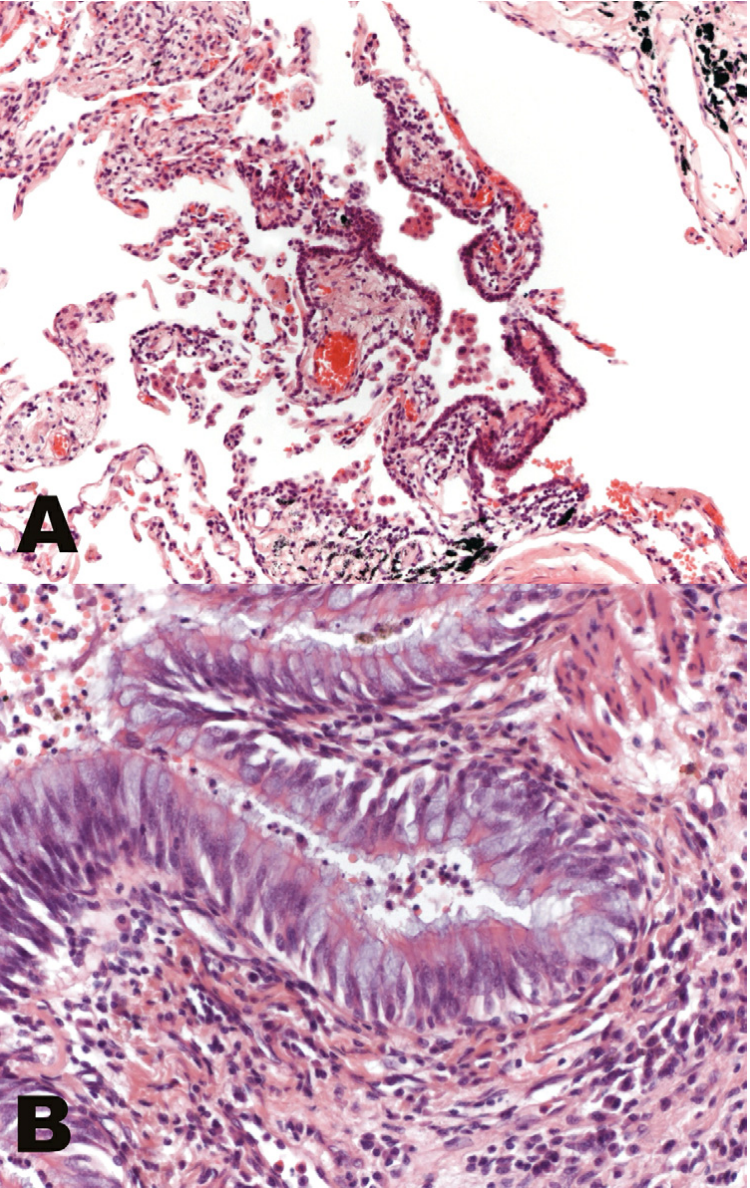
**Histology of non-alpha-1 antitrypsin deficiency emphysema. **In non-alpha-1 antitrypsin deficiency emphysema, chronic respiratory bronchiolitis, destruction of the alveolar architecture, prominent accumulation of macrophages (A, hematoxylin eosin, original magnification ×40) and marked bronchiolitis of the terminal bronchioles is found (B, hematoxylin eosin, original magnification ×100).

**Figure 3:**
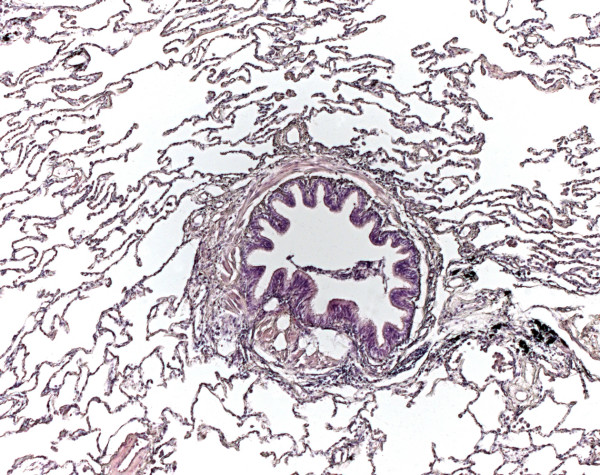
**Histology of normal lung tissue in patients with hamartochondroma. **In cases of hamartochondroma, only some macrophages and mucus can be detected in the bronchioli (hematoxylin eosin, original magnification ×15). No signs for emphysema or bronchiolitis could be detected.

The median value of age was 46.5 in AATD, 58.0 in non-AATD emphysema and 64.5 in hamartochondroma. The rate of females varied between 33.3% in AATD, 37.5% in non-AATD and 42.9% in hamartochondroma. Histology revealed destruction of the alveoli, intra-alveolar accumulation of macrophages, and acute alongside chronic bronchiolitis in all cases of AATD (Fig. 1A,1B) and non-AATD emphysema (Fig. 2A,2B) consistent with previous examinations. In cases of hamartochondroma only some macrophages and mucus in the bronchioli could be detected (Fig. 3).

### Transmission electron microscopy

**Figure 4:**
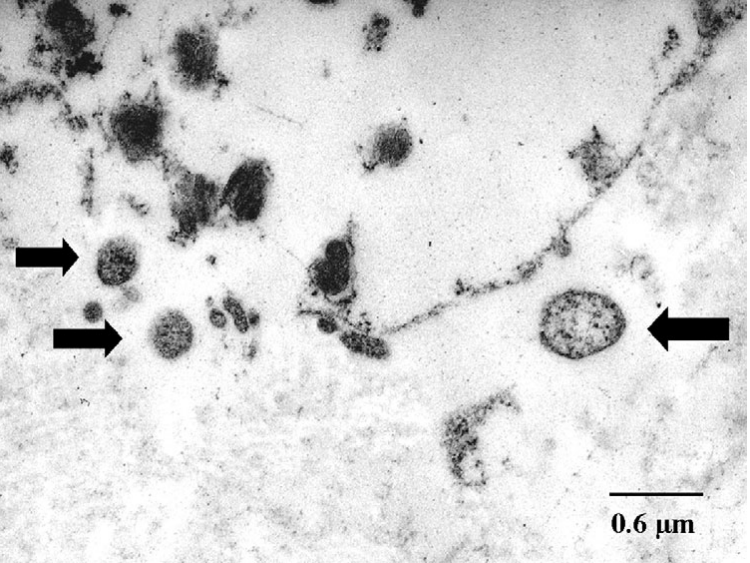
**Transmission electron microscopy of alpha-1 antitrypsin deficiency emphysema. **Chlamydial bodies (arrows) and destruction of the interstitial connective tissue are seen in alpha-1 antitrypsin deficiency. Ultrastructure is less well preserved after fixation in formaldehyde.

**Figure 5:**
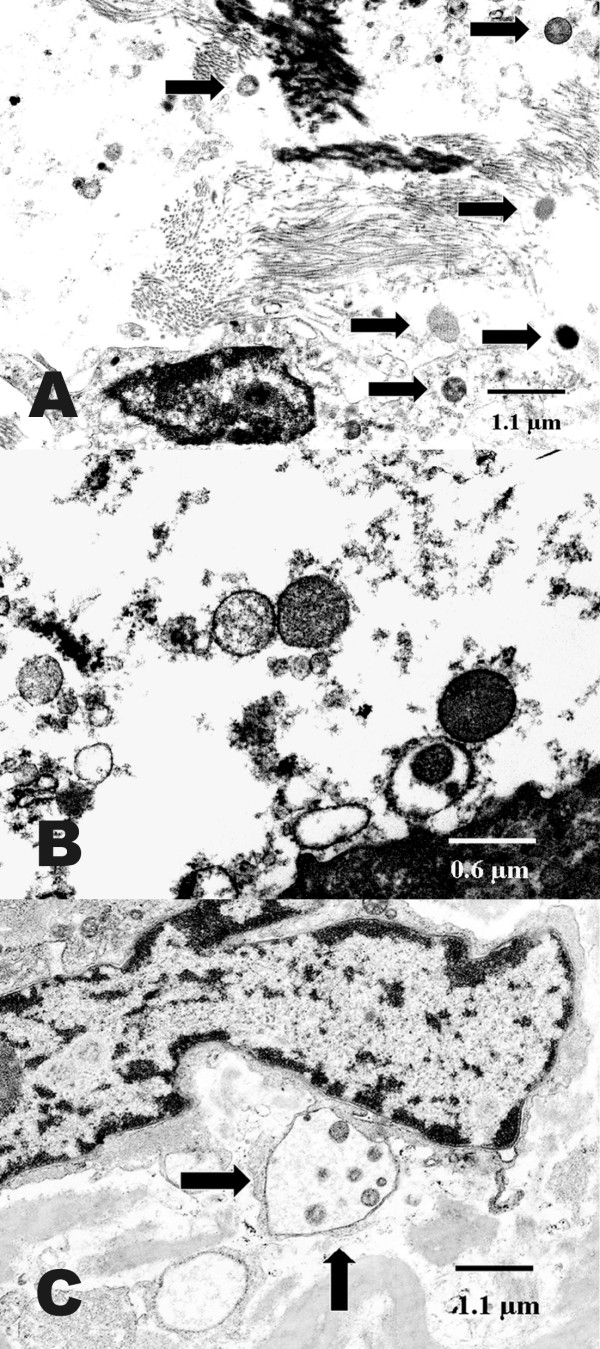
**Transmission electron microscopy of non-alpha-1 antitrypsin deficiency emphysema. **In non-alpha-1 antitrypsin deficiency destruction of the connective tissue and chlamydial bodies are detected (A-C). Higher magnification reveals different developmental stages of chlamydial bodies within a lytic area (B) and perinuclear inclusions (arrows) (C).

TEM images illustrate that the cell and tissue morphology appeared severely destroyed in emphysema samples. Chlamydial elementary and reticular bodies of 0.2 to 0.8 μm diameter were found on the surface of alveolar or bronchiolar epithelium and showed adherence to microvilli as previously described [16,17]. Reticular and some elementary bodies were seen in AATD (Fig. 4) and non-AATD emphysema (Fig. 5A,5B,5C), they were scattered within the interstitium (Fig. 5A) and also assembled in groups (Fig. 5B). Perinuclear inclusions could be detected in fibroblasts (Fig. 5C). Altogether, in 32 cases (82%) typical morphological structures indicating persistent chlamydial infection were present. In seven cases of emphysema, chlamydial bodies could not be detected or the findings were ambiguous. Detection rates were higher in AATD emphysema (5/5 = 100%) than in non-AATD emphysema (27/34 = 79.4%, Table 1). The control group of patients with hamartochondroma showed no signs of chlamydial infection in TEM.

**Table 1:** Detection of *Chlamydia spp. *in emphysema by TEM and PCR

**Groups**	**Cases**	***C. psittaci***	***C. pneumoniae***	**Fisher Yates test vs. controls**
**AATD emphysema**				
TEM	5	5 (100%)		0.00009
PCR	6	4 (66.7%)	1 (16.7%)	0.00310*
**Non AATD emphysema**				
TEM	34	27 (79.4%)		0.00000
PCR	31	9 (29.0%)	0 (0%)	0.03989
Controls				
TEM	14	0 (0%)		
PCR	14	0 (0%)	0 (0%)	

AATD = alpha-1 antitrypsin deficiency, * = for *C. psittaci *only

### Polymerase Chain Reaction (PCR)

**Figure 6:**
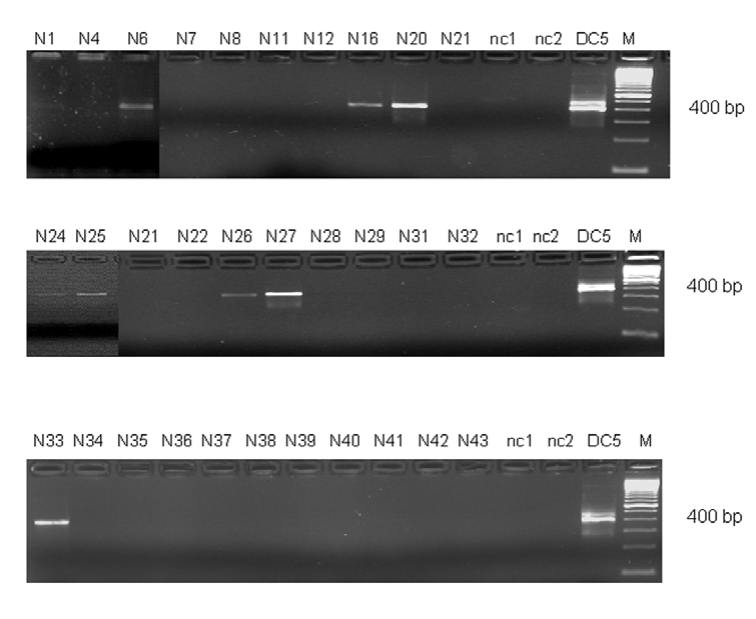
**Detection of chlamydiae by nested *omp *A-PCR from frozen lung tissue of patients with advanced emphysema (samples N1 to N43). **DNA was extracted from tissue samples, subjected to nested amplification, and PCR products were electrophoresed on 2% agarose gels. The amplicon of approximately 400 bp is specific for *Chlamydia psittaci*. Strain DC 5 of *Chlamydophila psittaci *was used as positive amplification control, nc1 and nc2 are negative (reagent) controls. Lane M shows the 100-bp ladder (Invitrogen, Karlsruhe, Germany).

Examination by PCR revealed the presence of *C. psittaci*-specific DNA in four (66.7%) specimens with AATD and nine (29%) with non-AATD emphysema (Fig. 6, Table 1). In one case of AATD, the amplicon was identified as *Cp. pneumoniae*. PCR was negative in all cases with hamartochondroma. The detection rate for *C. psittaci *in emphysematic tissue was higher from PET than from frozen material (50% vs. 21.9%, Fisher Yates test n. s.). In five cases, where both PET and frozen tissue were examined, one patient was positive in TEM and another one in PCR.

### DNA sequencing

**Figure 7:**
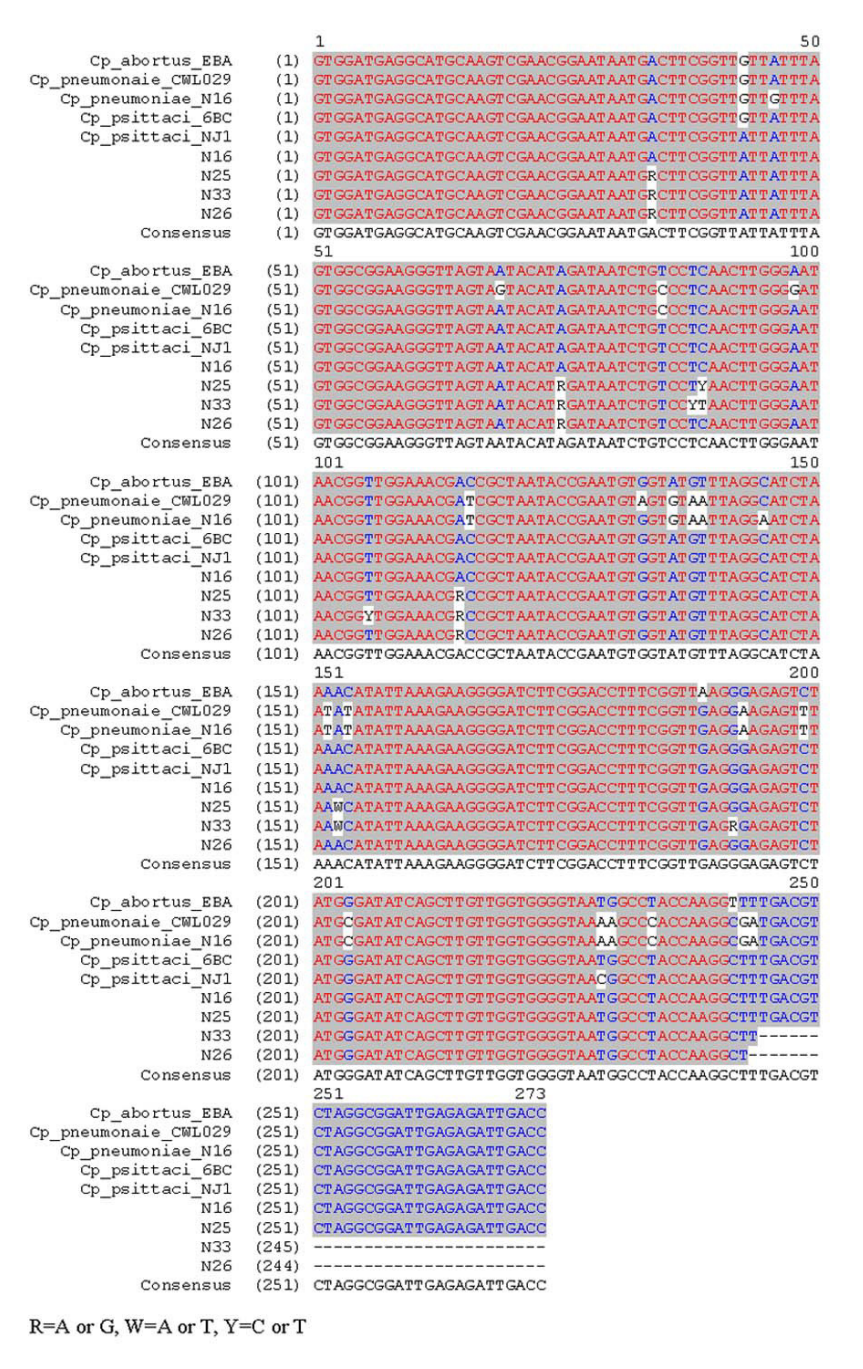
**Sequence alignment of four tissue samples and reference strains (16S signature region). **The samples N16, N25, N33, and N26 were sequenced in the 16S signature region. A BLAST search confirmed the species as *Cp. psittaci*.

To confirm the identity of the chlamydial species, DNA from four of the positive samples, i.e. N16, N25, N26, and N33, was sequenced in the 16S rRNA signature region (approximately 300 bp, Fig. 7). A BLAST search of these sequences revealed close to 100 % homology to the species *Cp. psittaci*.

## Discussion

Strains of *Cp. psittaci *are known to cause infections in over 130 avian species and 32 other domestic and wild animals. Classical psittacosis represents a systemic disease in psittacine birds of acute, protracted, chronic or subclinical manifestation. Avian strains of the agent are known to be pathogenic to humans, the symptoms being mainly non-specific and influenza-like, but severe pneumonia, endocarditis and encephalitis are not uncommon [24,25]. The possibility of persistent infection in man was first described in the 1950s [26].

In the present study and in previous investigations, transmission electron microscopy revealed elementary bodies as well as typical and aberrant reticular bodies, thus indicating active infection alongside persistent infection [10,16,17]. Chlamydiae could not be detected in each case, but the rate of positive findings in TEM and PCR (Table 1) was comparable to that of *Cp. pneumoniae *in atherosclerosis [27,28]. Pear-shaped elementary bodies as typically found in *Cp. pneumoniae *infection [29] were not observed. While the findings of TEM are more indicative of *Cp. psittaci *infection [30], it must be noted that this method provides no clear-cut differentiation among *Chlamydiaceae *species, for even strains of the same species exhibit different morphology at the various developmental stages. Rather unexpectedly, *Cp. pneumoniae *was detected only in one sample by PCR, not indicating an important role of this agent in the cases examined here. The fact that, apart from psittacosis, *Cp. psittaci *has not been associated with human respiratory disorders in recent decades may be a question of sensitivity and specificity of detection. Particularly PCR with its capability to specifically identify all individual species of *Chlamydiaceae *at a detection limit of less than one inclusion-forming unit has opened up new possibilities in this respect.

Higher detection rates of *Cp. psittaci *in tissue with histological evidence of inflammation in comparison to unselected frozen tissue (6/12 = 50% vs. 7/32 = 21.9%, Fisher Yates test n.s.) indicate an association with regional activity of infection and inflammation. Besides psittacosis, *Cp. psittaci *has been recently associated with chronic inflammation in patients with ocular adnexal lymphomas [31]. In COPD, activated macrophages and neutrophils produce matrix metalloproteinases which are relevant in the development of emphysema [32,33]. The release of matrix metalloproteinases was shown to be stimulated by cytokines produced in the course of chlamydial infection [34] and by chlamydial heat shock protein 60 as well [35]. These findings represent a link to the established pathogenetic concepts in pulmonary emphysema. *Cp. psittaci *was found at comparable and statistically significant rates in AATD and non AATD emphysema.

## Conclusions

The fact that the chlamydial agent present in the emphysema tissue was identified by DNA sequencing as an avian serovar of *Cp. psittaci *provides an important indication on the source of infection. It is conceivable that the patients were infected through contact with birds, although this could not be verified for lack of relevant data on history. The detection rate of chlamydiae in cases of AATD emphysema vs. non-AATD emphysema was clearly higher, thus indicating a relevant role of *Cp. psittaci *infection in this disorder, or at least a higher susceptibility of AATD patients for an infection of their lungs with *Cp. psittaci*. Further investigations concerning smokers and non-smokers, pathogenetic relevance and zoonotic implications are required. In any circumstances, *Cp. psittaci *has to be considered an underestimated pathogen with considerable importance in public health, although the various facets of its specific impact have yet to be evaluated.

## Competing interests

None declared.

## Authors' contributions

Dirk Theegarten has designed and organized this study, done light microscopy, written most of the manuscript, participitated in its statistical analysis and reviewed results of transmission electron microscopy. Olaf Anhenn participitated in designing the study, collected the data, performed statistical analysis and reviewed the manuscript. Helmut Hotzel carried out PCR analysis and DNA sequencing. Konrad Sachse has done the sequence alignments and written the parts of the manuscript concerning molecular biology and veterinary aspects, and also participated in PCR analysis. Mathias Wagner developed and used the tissue puncher for this study. Georgios Stamatis has done lung volume reduction surgery. Alessandro Marra and Georgios Stamatis have done the clinical parts. Alessandro Marra has reviewed the history of the patients. Grigori Mogilevski performed transmission electron microscopy. All authors have discussed and approved the final manuscript.

## Pre-publication history

The pre-publication history for this paper can be accessed here:


